# Supporting clinical research professionals through educational innovations

**DOI:** 10.3389/fphar.2023.1304415

**Published:** 2024-01-08

**Authors:** Diana Lee-Chavarria, Tammy L. Loucks, Rechelle Paranal, Royce Sampson, Carol Feghali-Bostwick

**Affiliations:** ^1^ South Carolina Clinical and Translational Research Institute, Medical University of South Carolina, Charleston, SC, United States; ^2^ Academic Affairs Faculty and Department of Obstetrics and Gynecology, Medical University of South Carolina, Charleston, SC, United States; ^3^ Office of Clinical Research, Medical University of South Carolina, Charleston, SC, United States; ^4^ Department of Psychiatry and Behavioral Sciences, College of Medicine, Medical University of South Carolina, Charleston, SC, United States; ^5^ Department of Medicine, College of Medicine, Medical University of South Carolina, Charleston, SC, United States

**Keywords:** micro-credential, digital badge, clinical research professional, research, training, professional development

## Abstract

Clinical Research Professionals (CRPs) are essential members of the Clinical and Translational Research Workforce. Many academic medical institutions struggle to recruit and retain these vital team members. One strategy to increase job satisfaction and promote the retention of CRPs is through educational initiatives that provide training and professional development. The South Carolina Clinical and Translational Research (SCTR) Institute Workforce Development (WD) team at the Medical University of South Carolina (MUSC) developed several trainings as part of our larger educational portfolio for CRPs. In 2022 WD implemented a digital badge micro-credential for SCTR’s Core Clinical Research Training (CCRT) course in collaboration with institution-wide education and technology offices. Beginning in January 2023, individuals were able to earn the CCRT Certified Digital Badge upon successful completion of the CCRT course.

## 1 Introduction

Clinical Research Professionals (CRPs) are essential members of the clinical and translational research workforce at Academic Medical Centers (AMCs). These professionals include clinical research coordinators, data managers, regulatory affairs specialists, clinical trial monitors, research nurses, and others (Sonstein and Jones, 2018; Knapke et al., 2022a). While the Principal Investigator (PI) has the final oversight of the study, many important tasks are often entrusted to CRPs as front-line workers. The role of a CRP has grown beyond solely participant management to encompass additional responsibilities including quality assurance, budgeting, regulatory compliance, database management, HIPAA compliance, and IRB submissions. CRPs also serve as the study’s central point of contact for research participants, clinicians, institutional research support offices, investigators, sponsors, and others (Speicher et al., 2012). The roles CRPs play in clinical research studies are both vast and essential.

Unfortunately, many AMCs struggle to recruit and retain these vital team members. There are various theories to explain challenges with CRP recruitment and retention, including compensation, a lack of professional recognition for their complex job functions, the absence of role-specific training and/or professional education, and expanding duties without the benefit of the previous two resources (Sonstein and Jones, 2018; Knapke et al., 2022b). As study protocols and regulations guiding research become more expansive, so do the roles of CRPs (Speicher et al., 2012). An increase in the breadth and scope of responsibility in the absence of additional training or job support can lead to job dissatisfaction and even burnout. Burnout may cause CRPs to depart from their roles or the entire clinical research workforce, leaving study teams ill-equipped to meet research study timelines and deliverables (Knapke et al., 2022a). A revolving door of novice CRPs can create a vacuum of institutional knowledge causing newly hired CRPs to learn on the fly, potentially slowing study efficiency and inadvertently jeopardizing compliance with study protocols, reporting, and regulatory requirements. The loss of experienced CRPs can have numerous ill effects on the conduct of clinical research at AMCs.

One strategy to address problems with CRP readiness and retention at AMCs are initiatives that provide job training and professional development to support this vital workforce. The SCTR Institute Workforce Development (WD) team develops and refines trainings as part of a large educational portfolio for all members of the research team. SCTR is the NIH-funded Clinical and Translational Science Award (CTSA) Hub at the Medical University of South Carolina (MUSC) and serves the entire state. CTSAs are funded by the National Center for Advancing Translational Sciences (NCATS) at the National Institutes of Health (NIH) to cultivate research from the laboratory into functional therapies for patient populations (National Center for Advancing Translational Sciences, 2023)^1^.

**FIGURE 1:**
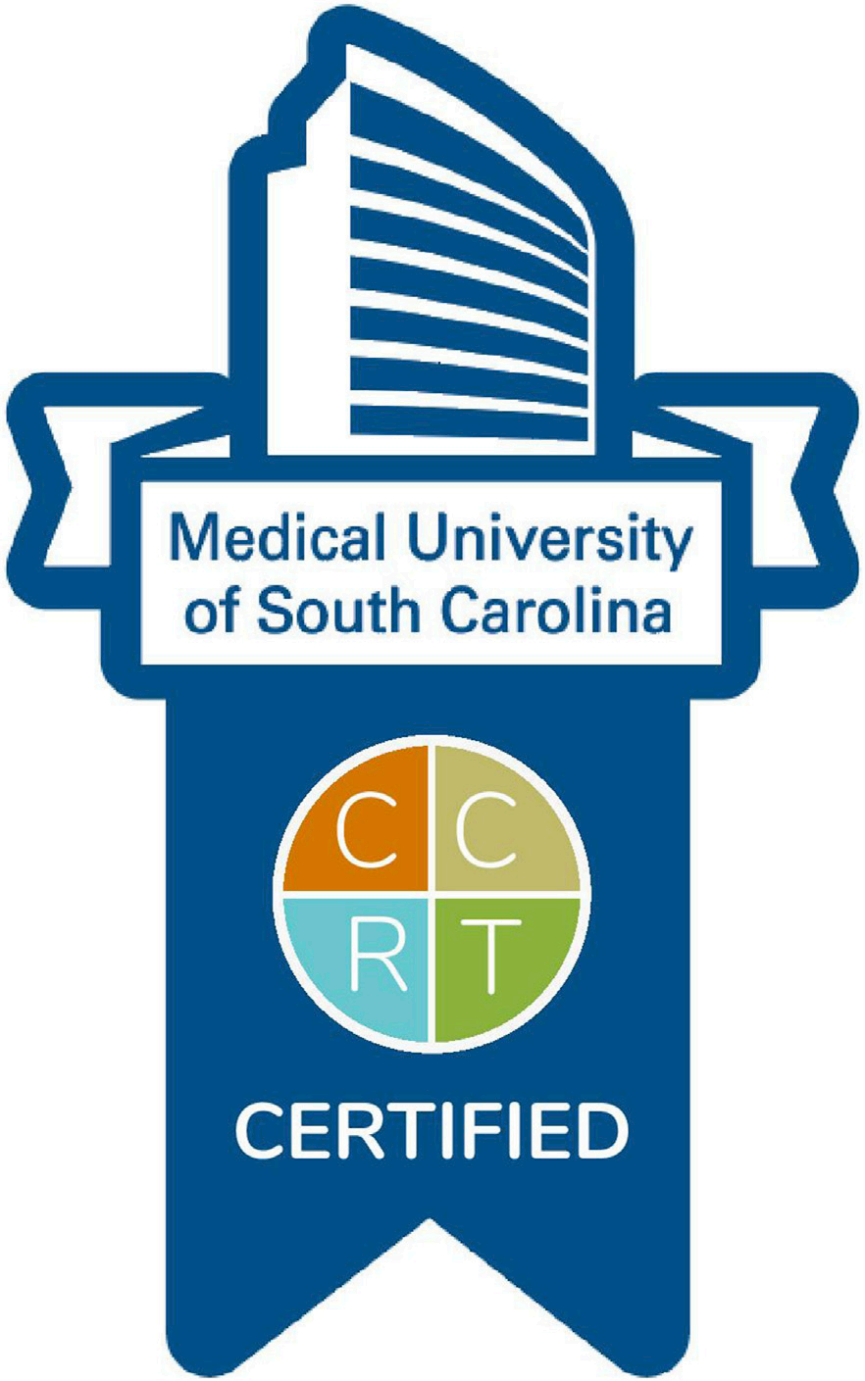
CCRT certified digital badge.

SCTR provides a portfolio of research training and professional development opportunities for staff, students, faculty, and investigators as part of its CTSA activities. Core Clinical Research Training (CCRT) is SCTR’s primary CRP training offering and has been embedded in the WD program for more than 10 years. CCRT provides foundational clinical research training for study team members to be effective in their jobs. CCRT focuses on the resources, processes, and regulations supporting clinical research conduct at MUSC. It is offered to MUSC employees and students who work in clinical research and collaborating institutions that partner with SCTR, including an associated Veteran’s Affairs (VA) Hospital. CCRT is considered a core element of training and orientation, and many new CRPs take the course as part of their introduction to conducting research at MUSC, although it is not part of mandatory onboarding training at the institution. SCTR WD debuted a digital badge for CCRT in 2023 [Figure 1].

Digital badges are micro-credentials in the form of an electronic symbol that document achievement or skills mastered through a specific training or coursework. Digital badges verify that the obtainer has achieved a certain level of knowledge and/or met a specific set of criteria to be awarded the badge (Stefaniak and Carey, 2019). Micro-credentials can be shared across digital platforms, including LinkedIn and social media, and on email signatures. Information about these required criteria, when the user completed the course/content, the date the badge was issued, and other relevant information from the issuer is embedded in an online platform and visible to outside users (Yu et al., 2015; Galindo, 2023).

This manuscript describes our experience to develop and obtain a digital badge micro-credential for the CCRT course. The CCRT Certified Digital Badge was predicated on the converging factors of new education technology resources at MUSC and the revision of CCRT’s content and format.

## 2 Opportunity

In 2019, the SCTR WD team conducted an institution-wide survey of research staff and faculty via REDCap to identify their research-related training needs and challenges. Respondents were asked about their preferences for learning environment (e.g., online, in-person), the value of specific research-focused learning topics (e.g., recruitment, research administration, research processes), and any barriers to utilizing existing trainings. More than half of respondents (52.9% of faculty and 58.0% of staff) reported a preference for attending online trainings (it should also be noted that this survey was conducted before the COVID-19 pandemic and implementation of remote work policies). Additionally, multiple responses indicated a need for additional training on internal and external research processes (Loucks et al., 2021).

Prior to 2020, CCRT included both in-person sessions (twice annually) and bi-monthly online sessions that were video recordings of presentations from the live course. Attendees received paper Certificates of Completion that were signed by the course director; these certificates were not able to be shared on digital platforms and did not verify achievement of skills but rather course participation. CCRT moved fully online during the COVID-19 pandemic to meet the needs of CRPs who were working remotely during that time. Considering the widespread adoption of remote work and feedback obtained from the 2019 survey, SCTR WD evaluated our existing portfolio and determined that an update of CCRT into a fully online, asynchronous course was necessary. During this same time frame, members of the WD team had been introduced to the idea of digital badging and MUSC was evaluating new learning management systems that could support a digital badge.

In 2022 SCTR WD began to refresh the existing course material and add new content with the goal of attaining a digital badge micro-credential for the course. As part of this refresh, all content and learning modules were revised. This initiative involved input from the SCTR regulatory staff and institutional offices, and an instructional designer who was versed in course design and adult learning principles. The instructional designer used Articulate 360 to create new modules for CCRT that were interactive, engaging, met MUSC’s digital accessibility requirements, and could be deployed using MUSC’s learning management system (LMS) for continuing education (Desire to Learn D2L, Brightspace).

The revised CCRT consists of 20 distinct modules and 15 required quizzes. These modules are broken down into Parts I-VI with relevant modules grouped together along the research project life cycle [Table 1]. Each instructional module begins with learning objectives and relevant terminology and acronyms. Modules are comprised of brief video segments followed by knowledge checks to allow the learner to reflect on the material they have learned and test their knowledge before moving to the next section. Depending on the number of subsections covered in each topic, there are three to six video segments each followed by a knowledge check. Modules are intended to be taken in the order which they are presented. The course is self-paced, and participants have 8 weeks to complete the course modules and quizzes. The course also includes a “Start Here” module that provides important information on how to navigate the learning management system.

**TABLE 1:** CCRT course modules.

Part	Module title	Learning objectives
I	Introduction to Core Clinical Research Training	• N/A
	SCTR Services	• explore resources available through SCTR Institute
		• distinguish between SCTR fee-based and free services
II	Evaluating Study Feasibility	• identify the key components of a comprehensive feasibility analysis
		• list the 3 self-service patient count tools that can be used to obtain patient count data for a feasibility assessment
		• identify the aspects of each component of a comprehensive feasibility analysis
	Recruitment Planning and Development	• name at least 3 factors that can influence recruitment strategies for a particular study
		• identify the web-based resources for recruitment that MUSC supports and the distinguishing features between them
		• list the ways MUSC patients can opt-out of research contact
		• identify best practices for recruitment messaging
	Inclusion of Special Populations in Research	• differentiate between the concepts of equality and equity
		• identify who are special populations in clinical and translational research
		• identify barriers to special populations participating in research
		• identify ways to integrate special populations into research
III	SPARCRequest	• review elements of the SPARCRequest system
		• describe how it is used in conducting research at MUSC
	Research Billing Compliance—Prospective Reimbursement Analysis (PRA)	• summarize the rationale for a PRA process
		• identify which studies require a PRA and which are exceptions
		• understand how to initiate and submit a PRA and PRA Amendment
	Understanding a Corporate Clinical Research Budget	• recognize the steps involved in the budgeting process
		• identify key components of a corporate clinical research budget
		• describe the importance of invoicing communication and how that results in money for the study team
	Overview of Epic	• describe EPIC and how it relates to research studies at MUSC
IV	Good Clinical Practice	• understand the errors previously made by investigators
		• name and understand the principles of the Belmont Report
		• understand the importance of regulations and guidelines in clinical research
	Institutional Review Board (IRB)	• define the role of the IRB
		• identify the types of IRB review
		• describe what information gets reviewed by the IRB
	Informed Consent and HIPAA	• differentiate between the different types of informed consent
		• describe the consent process including the key elements
		• explain HIPAA requirements in relation to research
V	Principal Investigator Roles and Responsibilities	• identify the regulatory bodies and guidelines that define PI responsibilities in the conduct of clinical trials
		• identify common problems in obtaining appropriate informed consent
		• distinguish to whom study activities may be delegated by the PI
	Regulatory Files	• determine what comprises regulatory files for research studies
		• identify the reasons why maintaining a regulatory binder is important to the success of your research study
		• identify organizational techniques that are beneficial for certain types of research and regulatory file organization
	Procedural Documentation for Clinical Research Operations	• identify the differences between Policies, SOPs, and MOPs
		• identify the benefits of developing SOPs, MOPs, and site-specific protocol plans
		• demonstrate basic knowledge of the 8-steps to write an SOP
	Investigational Drugs and Devices	• identify the FDA regulations regarding investigational drugs and devices
		• describe the institutional policies regarding studies utilizing investigational drugs
		• discuss best practices for managing investigational drugs
VI	Adverse Events, Protocol Deviations and Unanticipated Problems	• identify terms related to reportable events
		• differentiate between an AE and an SAE
		• apply reporting requirements related to safety reporting
	Overview of ClinicalTrials.gov	• identify the differences between ClinicalTrials.gov and the CT.gov Protocol Registration System
		• identify the purposes and beneficiaries of trial registration/results reporting
		• identify examples of the types of studies that need to be registered on CT.gov and report results
		• identify the timeframe in which a study needs to be registered on CT.gov per federal requirements and for ICMJE-affiliated journal publication
	Creating a Compliant Research Program	• examine ethical responsibilities and identify methods of reporting non-compliance activities
		• provide researchers and study teams with practical techniques to identify, monitor, and resolve compliance issues
		• demonstrate proactive behaviors to help safeguard against non-compliance
	Research Misconduct	• define what constitutes research misconduct
		• identify the steps MUSC takes toward preventing research misconduct
		• recognize the process for addressing an allegation of potential research misconduct
		• describe the protections affirmed for “whistleblowers"

There is no cost to participate, and personnel can self-enroll in the course through REDCap one of the four times a year it is offered—January, April, July, and October. Announcements about course registration are distributed through various institution-wide research-focused electronic newsletters and on the SCTR webpage.

Prerequisites include completing and passing the CITI MIAMI courses for Basic Human Research or Social and Behavioral Research and Good Clinical Practice and ICH prior to enrolling in CCRT. MUSC requires these courses to be taken by any personnel involved with the conduct of human subjects research prior to engaging in any research. CCRT builds on the foundations in the CITI courses and including these as a prerequisite also ensures that all participants have the same baseline level of knowledge before starting the course. Participants must complete all modules and receive an overall average of 80% or higher on the quizzes to earn the CCRT Certified Digital Badge.

### 2.1 Pedagogy

Both revised and new CCRT content was based around the process of conducting clinical research at MUSC. Content was also informed by the Joint Taskforce (JTF) for Clinical Trial Competency core competency domains (Multi-Regional Clinical Trials, 2023)^2^. The JTF competencies are widely accepted and broadly utilized across the CTSA consortium and the clinical research community. Two national organizations focused on the professional advancement of clinical research personnel, the Association of Clinical Research Professionals (ACRP) and the Society of Clinical Research Associates (SOCRA), have harmonized their training and certification exams to the JTF competencies (Sonstein and Jones, 2018).

### 2.2 Innovation

In 2022, MUSC initiated a new LMS with the capability to support digital badging, Brightspace by D2L, that had separate platforms for students and staff/professional development. CCRT and other non-credit courses were moved to the Endeavor platform on Brightspace and inherited the badging capability. The Brightspace/Endeavor platform can automatically issue digital badges to users who meet the set criteria. At the same time the MUSC Education Cabinet developed a digital badging policy that included a process to vet and award micro-credentials.

Taking into consideration this new institutional innovation and the ongoing revision of CCRT into a fully asynchronous online format, it was decided to proceed with implementing a digital badge for the course. The badge was developed in collaboration with institution-wide education and technology offices. The WD team, led by the SCTR Science Development Officer, prepared a proposal and sought approval from the MUSC Education Cabinet for the digital badge. An official application and the revised CCRT topics, instructors, and learning objectives were submitted for review as part of this proposal. Once the proposal was approved, the team worked with SCTR’s in-house graphic designer to create the badge’s visual element on an institutionally approved template that was established by the MUSC Brand Center in the Office of Communications and Marketing. Four options of badges were created, and the final design was selected by the full WD committee and then submitted to the MUSC Brand Center for approval. Once approved, the WD team worked with MUSC’s Office of Instructional Technology and Faculty Resources (ITFR) to add the badge to the course in the LMS and set the criteria for attainment. SCTR WD employs a full-time program coordinator who also collaborates regularly with the ITFR office to set the criteria for award dispensation and enroll and unenroll participants in the course.

Beginning in January 2023, individuals were able to earn the CCRT Certified Digital Badge upon successful completion of the CCRT course. Announcements about the inclusion of a digital badge in the updated CCRT course were made through the usual communications channels, including institutional research newsletters and on the SCTR website. The CCRT Certified Digital Badge is accredited and stored on the digital credentialing platform Canvas Credentials (formerly known as Badgr). Using Canvas Credentials, managers and other supervisory personnel can view the criteria required for obtaining the digital badge as well as evidence that the learner met the criteria.

## 3 Results

Between January and September 2023, 152 people registered for the CCRT course and 135 MUSC and associated VA personnel took the revised CCRT course. Participants’ “primary reason for taking the CCRT” is asked during course registration; 53.9% of registrants responded their primary reason was “Professional Development,” 34.2% stated that the course was required by their supervisor, and 5.9% responded that it was required by their training program. Participants’ “length of time in the field of research” was also collected during course registration; over half of respondents (57.1%) indicated that they had only been in research 12 months or less, with 34.2% noting that they had only been involved in research for 0–3 months. Of the 135 participants, 104 (77%) earned the CCRT Certified Digital Badge.

**FIGURE 2:**
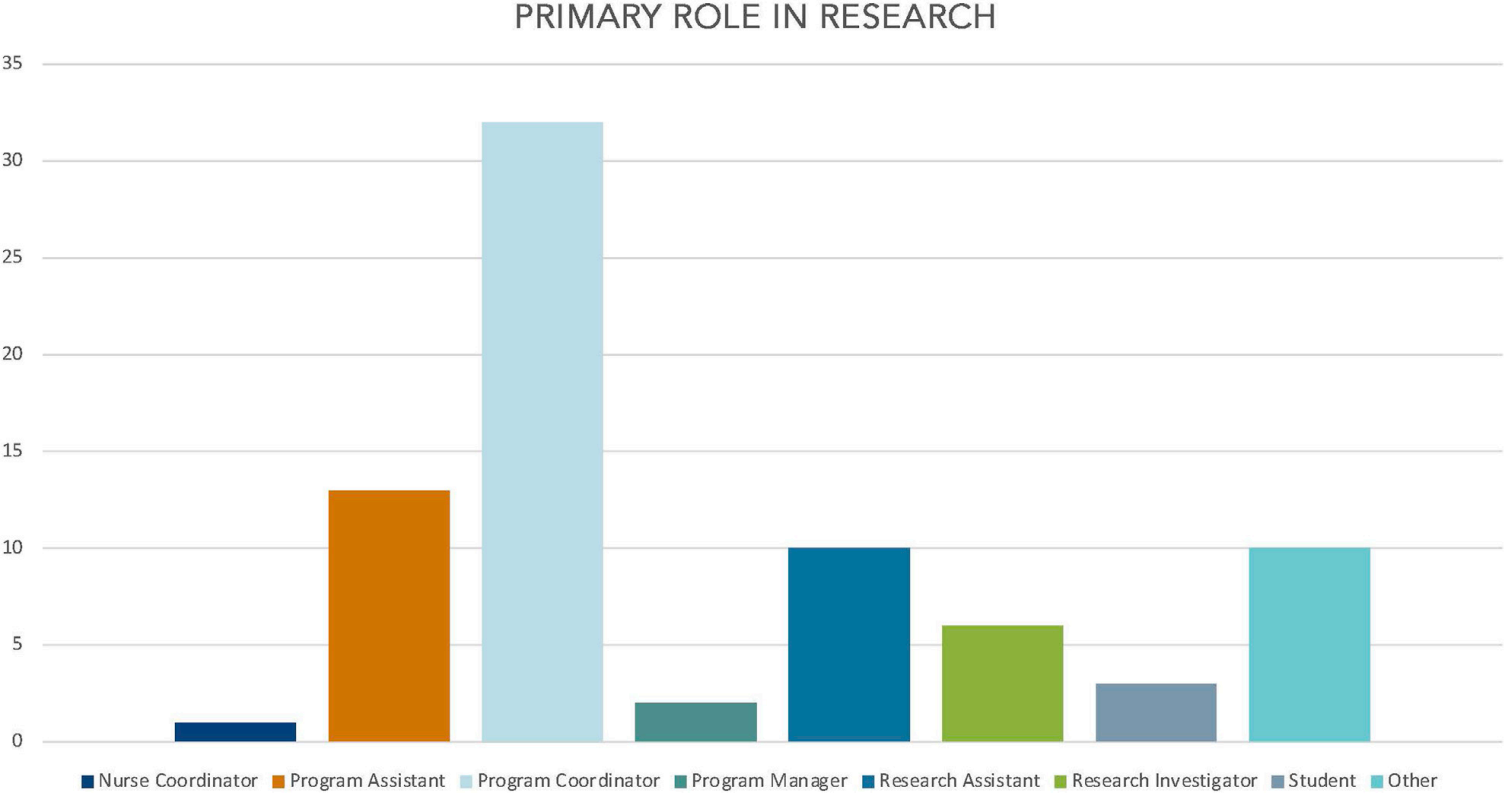
CCRT attendees—primary role in research.

CCRT course evaluations are conducted at the end of every 8-week cohort. Participants who successfully completed the course received a link to a REDCap survey to evaluate their overall training experience and provide input for continuous quality improvement. This survey is optional and confidential as no identifying data is collected, although respondents are asked to select their primary role in research. The majority (72%) of respondents from January-September 2023 (*n* = 77) self-identified as program coordinators (*n* = 32), program assistants (*n* = 13), or research assistants (*n* = 10) [Figure 2]. Participants are asked about their research experience, their overall thoughts on the course, and to provide input on future CCRT modifications.

**FIGURE 3:**
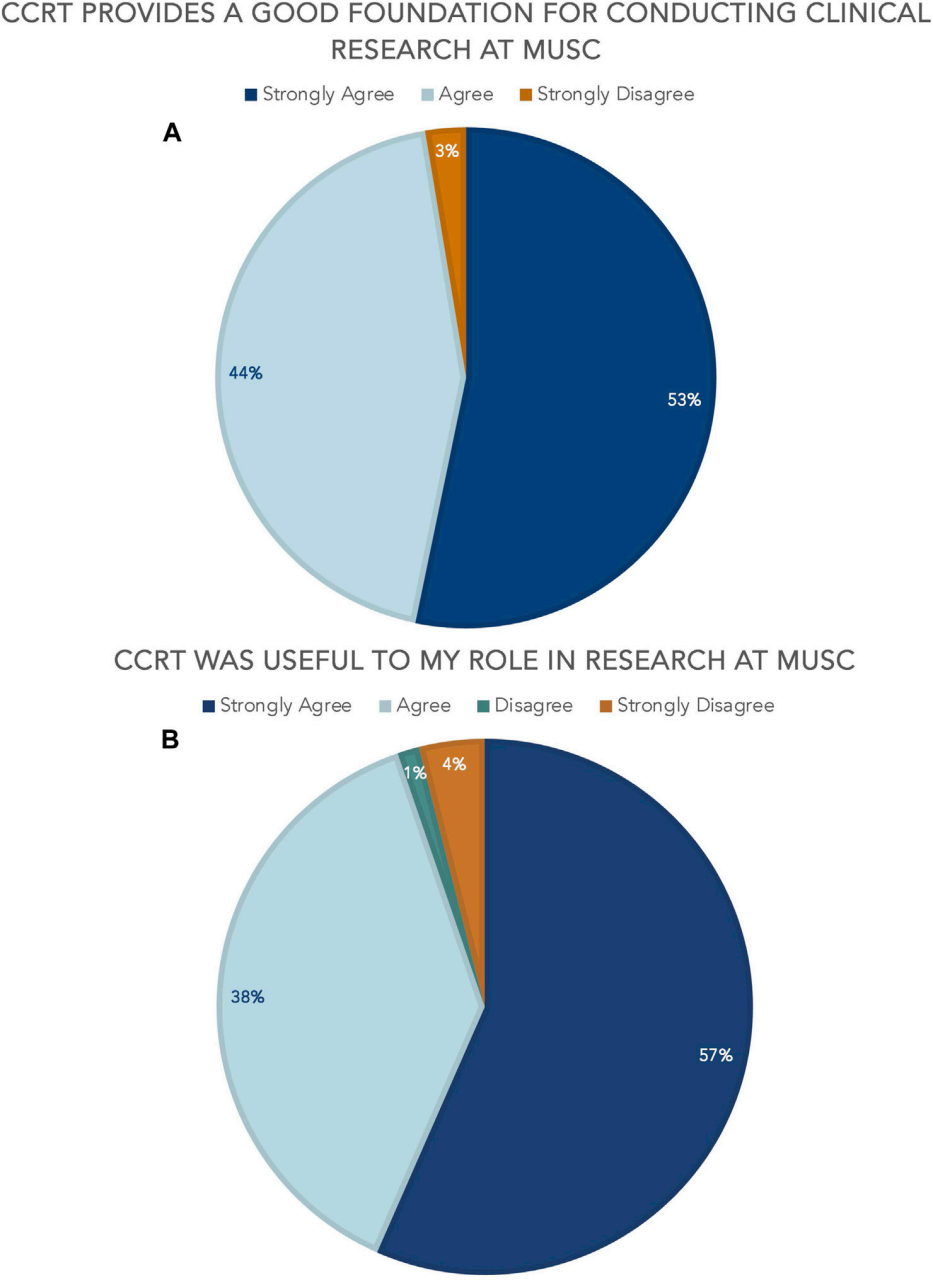
CCRT aggregate course evaluation results **(A)** CCRT provides a good foundation for conducting clinical research at MUSC **(B)** CCRT was useful to my role in research at MUSC.

Aggregate course evaluation results (*n* = 77) since January 2023 show the value of CCRT to the research learning environment at MUSC. 97% agree or strongly agree that CCRT provides a solid foundation for conducting clinical research at MUSC and 95% agree or strongly agree that CCRT was useful to their role in clinical research [Figure 3]. It is relevant to note that there are no questions specifically pertaining to the digital badge included in the overall course evaluation as this survey was developed prior to 2022. VA personnel provided feedback that some of the content was MUSC-focused and not directly relevant to them, but they still gave overall high marks to the course as a valuable learning opportunity.

When asked what they liked best about CCRT, numerous responses indicated the ability to complete the course at their own pace. This speaks to the importance of flexibility with online learning opportunities:

“The format of the course was easy to use and allowed for learning at one’s own pace.”

“Ease and flexibility in completing the course.”

“That it was self-directed.”

“It gave me a good foundation for conducting clinical research at MUSC.”

Responses indicated overwhelmingly that CCRT was perceived to be a valuable learning experience:

“The course was a good overview of clinical research at MUSC and provided a strong basis for a variety of research topics that are applicable to my job.”

“I think continuing education is helpful in minimizing mistakes.”

“Being that I’m new in research, I feel like by taking this course I have a great foundation on the research sector, and I will be able to continue to build upon that foundation as I learn and grow more professionally.”

“CCRT breaks down the core development that is needed to succeed in this role.”

Moving forward, SCTR’s Evaluation and Quality Improvement (EQI) team will conduct a focused evaluation on the CCRT Certified Digital Badge. They will convene a focus group for a one-year follow up to gather data on the value of the digital badge from CCRT participations who have obtained the micro-credential. This will begin in early 2024 to facilitate data collection 1 year after the first cohort received their badges and will enable the evaluators to get a better sense of how the badge was used and/or perceived as beneficial. EQI also plans to do six-month follow-up surveys beginning in 2024 to continuously gather quality improvement data for the badge. These surveys will include questions about the prior awareness and anticipated incentive of the CCRT digital badge, the overall perceived value and utilization of the badge, and applicability of the badge to participants’ current roles at MUSC. These plans will also allow the team to identify how many participants—stratified by primary research position—are still at MUSC. As digital badging becomes more established at the institution, it may also be useful to add a question to the course evaluation asking if the digital badge was a factor in taking and/or completing the course. Additionally, SCTR WD and EQI are exploring ways to share this strategy and the opportunity to earn the CCRT Certified Digital Badge with our state-wide collaborators to increase interest and engagement in CRP career development.

## 4 Discussion

There is a general acceptance that CRPs are integral to the conduct of clinical research, and “provision of adequate training and support to the research coordinator is critical to the overall goal of human subject protection at a given institution” (Speicher et al., 2012). CRPs serve numerous vital roles in the conduct of clinical research studies, both patient-facing and behind the scenes. One global survey of CRPs conducted in 2014 found that the increased job complexities and responsibilities of clinical research personnel requires additional skills (Sonstein and Jones, 2018). However, increasing responsibilities without commensurate skills training is not a sustainable practice and could lead to adverse study outcomes. In addition, a 2008 study found that 42% of CRPs surveyed worked more than their scheduled 40-h/week completing their study tasks (Speicher et al., 2012). Results such as these make it easier to understand how a CRP could feel overextended without time to pursue continuing education or job training and underappreciated in their roles.

The increasing breadth and depth of their roles, combined with inadequate role-specific training and professional recognition, have contributed to problems with CRP recruitment and retention. These are not the only issues affecting CRP careers, but the only addressed in this manuscript; factors such as wages and job flexibility are not always easy to address and can be dependent on institution or state policies (Knapke et al., 2022a). While the authors do not suggest that educational innovations such as digital badges can solve all issues around CRP job satisfaction and retention, we do feel that they are one tool that can be used to support employees. This idea appears to be gaining acceptance in the clinical research workforce; ACRP, an organization dedicated to CRP advancement, awards a digital badge to those who obtain certification (Association of Clinical Research Professionals, 2017)^3^. Attaching digital badges to courses used as foundational training for CRPs is one step towards recognizing the body of knowledge and scope of practice required for CRPs at AMCs.

Digital badges have several advantages for both AMCs and CRPs. Online training with the inclusion of a sharable, verified micro-credentials can provide validation of the standards met and skills achieved that can be shared both inside and outside of the institution (Stefaniak and Carey, 2019; Galindo, 2023). This is responsive to an issue identified from an evaluation conducted as part of the “Collaborative Conversations” Un-meeting series in November and December 2020; CRPs reported problems in demonstrating competency and recording completed certifications and trainings (Knapke et al., 2022b). First, badges enable employees to have a permanent and visible record of skills attained to demonstrate their professional achievement and career development. Second, employees can build their professional online presence through the ability to share the micro-credential on sites such as LinkedIn or on professional e-portfolios (Pakstis, 2019)^4^. Similarly, the inclusion of badges on email signatures allows for broader distribution of the accomplishment than could be achieved by a paper certificate of completion. Third, digital badges serve as a visual token of skill attainment rather than merely course participation. The promise of a tangible reward may provide an incentive for taking and/or completing trainings (Yu et al., 2015), especially if the CRP has little free time and must choose carefully between continuing education opportunities.

The inclusion of digital badges in continuing education and/or training content may also substantiate the institution’s commitment to their employees and an interest in supporting their professional development. Theoretically, an employee who is recognized for their achievements may be more motivated to stay in their role which could enforce recruitment and retention (Pakstis, 2019)^4^. The use of digital badges can also reduce the administrative burden of managing a course. The time needed to verify a learner’s scores, ensure they completed all requirements, create a personalized certification of completion (or similar), and send the certificate to each learner (via email or regular mail) can be time-consuming. Because of time constraints, training opportunities for professional development that are not in traditional credit courses may not receive any type of formal certification of completion (Yu et al., 2015). This administrative burden is alleviated by the automated processes involved in issuing electronic micro-credentials and providing learners with a digital badge, allowing CRPs and others to demonstrate competency even in courses targeted at professional development.

It is apparent that continuous learning is necessary as job complexities increase and new innovations arise. CRPs and those responsible for the conduct of studies must be able to prove their competence and knowledge around these issues. Continued fluency in new skills is compulsory for career advancement and to support CRP professional development (Pakstis, 2019)^4^. As previously discussed, one solution to these concerns is continuing education and training that is accessible to CRPs. In this terminology, “accessible” means being available in both a place and time convenient for CRPs; training that cannot be taken is not useful for anyone. Learning and professional development opportunities must be applicable to the participants’ roles, or they will not find it beneficial. SCTR WD was thoughtful in revisioning CCRT to make it as accessible to CRPs as possible. This includes the course being asynchronous and online to enable CRPs to complete the work at the times that best work for them, as well as eliminating course fees to prevent financial barriers. It is reasonable to conclude that incorporating a digital badge into a course that is broadly utilized by CRPs would be valuable to the same group. When deciding where to integrate a digital badge, the WD team felt confident in selecting CCRT since evaluation responses show that the course has a high perceived value. The CCRT Certified Digital Badge recognizes CRPs at the enterprise who have attained foundational knowledge in conducting compliant clinical research.

The broad acceptance of online learning has increased the opportunity for micro-credentialing and was vital in our digital badge development. Beginning in 2019, MUSC acquired four hospitals in various parts of South Carolina. These sites did not have a robust clinical research infrastructure, but planned to start conducting research after affiliating with MUSC. Online learning such as CCRT enable CRPs at both the main campus in Charleston and the regional hospitals in rural areas across the state to access continuing education and training opportunities. Another factor that was vital to the development of the CCRT Certified Digital Badge was the addition of an instructional designer who was versed in online learning principles to the WD team. Their expertise enabled us to move from a video recorded presentation format and create new interactive and engaging learning modules to improve the online learning experience.

The authors also wish to acknowledge some constraints on the outcome responses. First, the data is from a small sample size of CRPs (*n* = 77) and only a small percentage of CRPs at the institution have received a badge thus far. Additionally, the revised course with the digital badge has only been active since January 2023, so we do not have longitudinal data showing the impact/effects of the badge. This is why continuous quality improvement evaluations and focus groups will be important; focus groups will begin in January 2024.

## Data Availability

The raw data supporting the conclusion of this article will be made available by the authors, without undue reservation.

## Footnotes

1
https://ncats.nih.gov/ctsa

2
https://mrctcenter.org/clinical-trial-competency/framework/domains/

3
https://acrpnet.org/wp-content/uploads/dlm_uploads/2017/04/Digital-Badging-FAQs-1.pdf

4
https://www.harvardbusiness.org/for-organizations-and-learners-the-benefits-of-badging-are-clear/
