# Supplementary material for: Supporting clinical research professionals through educational innovations
Source: Front Pharmacol. 2024 Jan 8;14:1304415. doi: 10.3389/fphar.2023.1304415 (PMC10800862; doi:10.3389/fphar.2023.1304415)
Supplement: Supplementary file 1 [file DataSheet1.PDF]

Supplementary Material

Supporting Clinical Research Professionals through Educational Innovations

Diana Lee-Chavarria[1*], Tammy L. Loucks[1,2], Rechelle Paranal[1], Royce Sampson[1,3,4], Carol Feghali-Bostwick[5]

[1]South Carolina Clinical and Translational Research Institute, Medical University of South Carolina, Charleston, SC, United States

[2]Academic Affairs Faculty and Department of Obstetrics and Gynecology, Medical University of South Carolina, Charleston, SC, United States

[3]Office of Clinical Research, Medical University of South Carolina, Charleston, SC, United States

[4]Department of Psychiatry and Behavioral Sciences, College of Medicine, Medical University of South Carolina, Charleston, SC, United States

[5]Department of Medicine, College of Medicine, Medical University of South Carolina, Charleston, SC, United States

*** Correspondence:** Diana Lee-Chavarria leeachar@musc.edu

Supplementary Figure 1. CCRT Overall Course Evaluation, pages 1-2

CCRT Overall Course Evaluation

Please review the following questions to evaluate your overall experience with the Core Clinical Research Training course. Your responses will be used to help improve the quality of future trainings.

Please select today's date. ____________________

Please indicate your primary role related to research

- ○ Nurse Coordinator
- ○ Program Assistant
- ○ Program Coordinator
- ○ Program Manager
- ○ Research Administrator
- ○ Research Analyst/Data Manager
- ○ Research Assistant
- ○ Research Investigator
- ○ Research Specialist
- ○ Student
- ○ Other

If you answered other, please describe your primary role in research. ____________________

Please tell us about your research experience.

	0-3 months	3-6 months	6-12 months	1-3 years	3-6 years	>6 years
How long have you been in your current role at MUSC?	○	○	○	○	○	○
How long have you been involved in the conduct, support, or administration of clinical research?	○	○	○	○	○	○

Please provide your overall assessment of the Core Clinical Research Training (CCRT) course by indicating your level of agreement with the following statements.

	Strongly Disagree	Disagree	Agree	Strongly Agree
CCRT provided a good foundation for conducting clinical research at MUSC.	○	○	○	○
CCRT was well-organized.	○	○	○	○
CCRT is a learning opportunity that I would recommend to a colleague.	○	○	○	○
CCRT was useful to my role in research at MUSC.	○	○	○	○

What is the primary reason for your rating to the previous question? ____________________

Please take a few minutes to provide your feedback to help us improve CCRT.

What did you like best about this course?

____

What did you like least about this course?

____

Please select any modules that you feel would benefit you if offered as additional instruction (select all that apply)

- ☐ Evaluating Study Feasibility
- ☐ Recruitment Planning and Development
- ☐ Inclusion of Special Populations in Research
- ☐ SPARC Request
- ☐ Research Billing Compliance - Prospective Reimbursement Analysis (PRA)
- ☐ Understanding a Corporate Clinical Research Budge[illegible]
- ☐ Good Clinical Practice
- ☐ Institutional Review Board (IRB)
- ☐ Informed Consent and HIPAA
- ☐ Principal Investigator (PI) Roles and Responsibilities
- ☐ Regulatory Files
- ☐ Procedural Documentation for Clinical Research Operations
- ☐ Investigational Drugs and Devices
- ☐ Adverse Events, Protocol Deviations, and Unanticipated Problems
- ☐ Creating a Compliant Research Program
- ☐ Research Misconduct
- ☐ Overview of ClinicalTrials.gov
- ☐ Overview of Epic
- ☐ SCTR Services

Are there other topics that you would recommend be included in future CCRT courses?

____
